# Selective CDK9 inhibition overcomes TRAIL resistance by concomitant suppression of cFlip and Mcl-1

**DOI:** 10.1038/cdd.2013.179

**Published:** 2013-12-20

**Authors:** J Lemke, S von Karstedt, M Abd El Hay, A Conti, F Arce, A Montinaro, K Papenfuss, M A El-Bahrawy, H Walczak

**Affiliations:** 1 Centre for Cell Death, Cancer and Inflammation, UCL Cancer Institute, University College London, 72 Huntley Street, London WC1E 6DD, UK; 2 Clinic of General and Visceral Surgery, University of Ulm, Albert-Einstein-Allee 23, 89081 Ulm, Germany; 3 Department of Experimental Oncology and Molecular Medicine, Fondazione IRCCS Istituto Nazionale dei Tumori, 20133 Milan, Italy; 4 Cancer Immunology Unit, University College London, 72 Huntley Street, London WC1E 6DD, UK; 5 Department of Histopathology, Imperial College London, Du Cane Road, London W12 0NN, UK

**Keywords:** CDK9, TRAIL, NSCLC, PIK-75, SNS-032

## Abstract

Tumor necrosis factor-related apoptosis-inducing ligand (TRAIL) can induce apoptosis in many cancer cells without causing toxicity *in vivo.* However, to date, TRAIL-receptor agonists have only shown limited therapeutic benefit in clinical trials. This can, most likely, be attributed to the fact that 50% of all cancer cell lines and most primary human cancers are TRAIL resistant. Consequently, future TRAIL-based therapies will require the addition of sensitizing agents that remove crucial blocks in the TRAIL apoptosis pathway. Here, we identify PIK-75, a small molecule inhibitor of the p110*α* isoform of phosphoinositide-3 kinase (PI3K), as an exceptionally potent TRAIL apoptosis sensitizer. Surprisingly, PI3K inhibition was not responsible for this activity. A kinome-wide *in vitro* screen revealed that PIK-75 strongly inhibits a panel of 27 kinases in addition to p110*α*. Within this panel, we identified cyclin-dependent kinase 9 (CDK9) as responsible for TRAIL resistance of cancer cells. Combination of CDK9 inhibition with TRAIL effectively induced apoptosis even in highly TRAIL-resistant cancer cells. Mechanistically, CDK9 inhibition resulted in downregulation of cellular FLICE-like inhibitory protein (cFlip) and Mcl-1 at both the mRNA and protein levels. Concomitant cFlip and Mcl-1 downregulation was required and sufficient for TRAIL sensitization by CDK9 inhibition. When evaluating cancer selectivity of TRAIL combined with SNS-032, the most selective and clinically used inhibitor of CDK9, we found that a panel of mostly TRAIL-resistant non-small cell lung cancer cell lines was readily killed, even at low concentrations of TRAIL. Primary human hepatocytes did not succumb to the same treatment regime, defining a therapeutic window. Importantly, TRAIL in combination with SNS-032 eradicated established, orthotopic lung cancer xenografts *in vivo*. Based on the high potency of CDK9 inhibition as a cancer cell-selective TRAIL-sensitizing strategy, we envisage the development of new, highly effective cancer therapies.

## Introduction

*De novo* and acquired resistance to conventional chemotherapy remains the major obstacle in treating many cancers today. Intrinsic apoptosis resistance of cancer cells often involves disabling of the intrinsic apoptotic machinery.^1^ Therefore, targeting cancer cells via the extrinsic cell death machinery involving death receptors of the tumor necrosis factor (TNF) superfamily has become an attractive approach in cancer research. However, attempts to use cell death-inducing CD95L or TNF for systemic therapy were hampered by severe toxicity.^2, 3^ In contrast, TNF-related apoptosis-inducing ligand (TRAIL) can induce apoptosis selectively in tumor cells *in vitro* and *in vivo.*^4, 5^

Based on these findings, TRAIL-receptor (TRAIL-R) agonists, comprising recombinant soluble TRAIL and agonistic TRAIL-R antibodies, are currently evaluated in clinical trials. However, so far these trials only showed very limited therapeutic benefit.^6^ It has emerged that, although TRAIL is capable of inducing apoptosis in many cancer cell lines *in vitro* and *in vivo*, about 50% of cancer cell lines and the majority of primary tumor cells are TRAIL resistant.^7^ The limited success of clinical trials conducted so far is likely to be attributable to this fact. However, combinatorial treatment with sensitizing agents can break TRAIL apoptosis resistance resulting in synergistic and selective killing of tumor cells.^4^ These findings have encouraged extensive research into identifying potent TRAIL-sensitizing agents that do not sensitize non-transformed cells.

Binding of TRAIL to cognate apoptosis-inducing TRAIL-R1 (DR4)^8^ and/or TRAIL-R2 (DR5)^9^ results in receptor trimerization. The adaptor protein FAS-associated protein with death domain (FADD) is recruited to the death domain (DD) of trimerized TRAIL-Rs and, in turn, enables caspase-8 and -10 recruitment to and activation at the death-inducing signaling complex (DISC).^10, 11, 12, 13, 14^ In type-I cells, activation of caspase-8 and -10 at the DISC results in sufficient activation of the effector caspase-3, ultimately resulting in apoptosis. In type-II cells, additional activation of the mitochondrial pathway is required to neutralize X-linked inhibitor of apoptosis protein (XIAP)-mediated effector caspase inhibition via release of Smac/DIABLO from mitochondria.^15^

In order to prevent excessive apoptosis induction by TRAIL, several mechanisms that negatively regulate the TRAIL apoptosis pathway have evolved that are frequently exacerbated by cancer cells. The cellular FLICE-like inhibitory protein (cFlip) competes with caspase-8 for binding to FADD, thereby preventing caspase-8 activation and, consequently, apoptosis induction.^16^ Other cellular factors that antagonize apoptosis induction by TRAIL include the inhibitor of apoptosis proteins (IAPs).^17^ Among these, XIAP has been shown to have a major role in mediating resistance to TRAIL-induced apoptosis.^18^ In type-II cells, resistance to TRAIL-induced apoptosis can be mediated by high expression of anti-apoptotic Bcl-2 family members such as Bcl-2, Bcl-xL and Mcl-1 that antagonize truncated Bid-triggered Bax/Bak-mediated mitochondrial outer membrane permeabilization and the consequent release of the pro-apoptotic factors cytochrome *c* and Smac/DIABLO.^19^

Kinase inhibitors have emerged as a novel class of targeted small molecule agents with great therapeutic potential in cancer treatment. This is owed to the fact that kinases are crucial components of most cellular signaling pathways that promote tumor cell survival, growth, migration, invasion and metastasis. Several inhibitors of the phosphoinositide-3 kinase (PI3K) pathway are currently in clinical trials^20^ and, interestingly, pan-PI3K inhibitors, inhibiting all four catalytic isoforms (p110*α*, *β*, *γ* and *δ*), have been shown to sensitize to TRAIL-induced apoptosis.^21, 22^ Activating mutations of the *α*-isoform of PI3K (p110*α*) occur with frequencies of up to 30% in cancer^23^ and, recently, mutated p110*α* was suggested to render cancer cell lines resistant to TRAIL-induced apoptosis.^24^ Therefore, we set out to test whether specific inhibition of p110*α* would render cancer cells sensitive to TRAIL-induced apoptosis.

## Results

### The p110*α* inhibitor PIK-75 potently sensitizes tumor cells to TRAIL-induced apoptosis independently of PI3K inhibition

**Figure 1:**
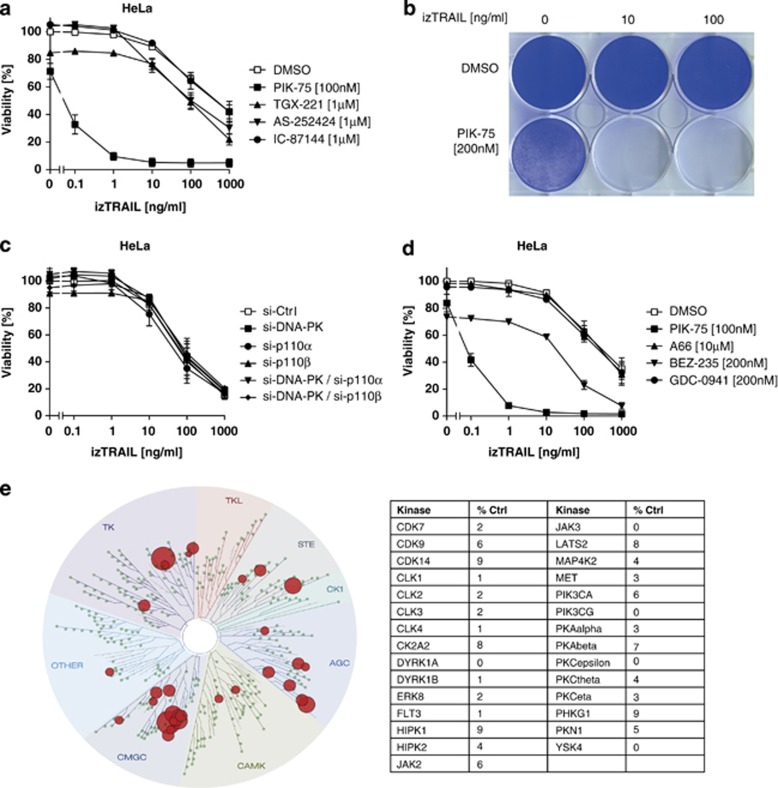
PIK-75 profoundly sensitizes cancer cells to TRAIL-induced apoptosis independently of PI3K inhibition. (**a**) HeLa cells were preincubated for 1 h with the indicated PI3K inhibitors and subsequently stimulated with izTRAIL at the indicated concentrations. Cell viability was quantified after 24 h. (**b**) A549 cells were treated with DMSO or PIK-75 (200 nM) for 1 h and subsequently stimulated with izTRAIL for 24 h. Long-term survival was visualized after 7 days by crystal violet staining. One of two independent experiments is shown. (**c**) HeLa cells were transfected with the indicated siRNAs. After 48 h, cells were stimulated with izTRAIL at different concentrations. Cell viability was analyzed 24 h later. (**d**) HeLa cells were preincubated for 1 h with the different PI3K inhibitors at the indicated concentrations and subsequently stimulated with izTRAIL at different concentrations. Cell viability was quantified after 24 h. (**e**) The capacity of PIK-75 at 200 nM to bind to a panel of 451 human kinases was determined by analyzing the binding interaction (%) compared with DMSO (=100%) using Kinomescan. Hits (<10% remaining activity) are visualized (red circles) and listed in the table. Values (**a**, **c** and **d**) are means±S.E.M. of three independent experiments

To investigate whether inhibition of one of the PI3K isoforms is sufficient to sensitize cancer cells to TRAIL-induced apoptosis, we treated HeLa cells with TRAIL in the presence or absence of pharmacological inhibitors that have been reported to be isoform specific (PIK-75 (p110*α*), TGX-221 (p110*β*), AS-252424 (p110*γ*) and IC-87114 (p110*δ*)) (for IC50 values see Supplementary Figure S1a). Whereas co-treatment with inhibitors of the *β*-, *γ*- and *δ*-isoforms of PI3K showed only marginal effects, co-treatment with PIK-75 profoundly increased TRAIL sensitivity of HeLa cells shifting the sensitivity of these cells by 3–4 orders of magnitude (Figure 1a and Supplementary Figure S1b). HeLa cells are sensitive to higher concentrations of TRAIL; however, many other cancer cell lines and most primary cancer cells are TRAIL resistant.^7^ Therefore, we next tested whether the exceptionally potent TRAIL sensitization exerted by PIK-75 in HeLa cells would translate into sensitization of the highly TRAIL resistant non-small cell lung cancer (NSCLC) cell line A549. Indeed, when treated with PIK-75 A549 cells became sensitive to apoptosis induction by TRAIL, even at concentrations of TRAIL as low as 10 ng/ml (Supplementary Figure S1c). Intriguingly, when examining clonogenic survival, we observed that this novel combination almost completely obliterated clonogenic survival of A549 cells (Figure 1b).

Having shown that PIK-75, a potent inhibitor of p110*α*, is a very effective TRAIL sensitizer, we next investigated whether specific inhibition of the p110*α* isoform of PI3K was capable of breaking TRAIL resistance in cancer cells and, hence, responsible for the PIK-75-mediated effect. To this end, we performed RNAi-mediated silencing of p110*α* as compared to p110*β* and DNA-PK, which has been shown to be inhibited by PIK-75 in addition to p110*α*.^25^ Surprisingly, silencing of p110*α*, p110*β* and DNA-PK, or any combination thereof, did not sensitize HeLa cells to TRAIL-induced apoptosis (Figure 1c, knockdown efficiency in Supplementary Figure S1d). In order to test the possibility that very low amounts of protein remaining after knockdown may be sufficient to maintain resistance, we also used two pan-PI3K inhibitors, GDC-0941 and BEZ-235, which both inhibit p110*α* with even lower IC50s than PIK-75.^26, 27^ In addition, we also used A66, a novel p110*α*-specific inhibitor^28^ (for IC50 values see Supplementary Figure S1e). However, when testing these three compounds, we found that none of them reproduced the extent of sensitization observed with PIK-75 co-treatment (Figure 1d). Interestingly, BEZ-235 was more efficient than PIK-75 at suppressing PI3K activity as assessed by phosphorylation of AKT (Supplementary Figure S1f). Moreover, concentrations of up to 10 *μ*M of A66 were not able to suppress pan-PI3K activity in HeLa cells, which have been reported to harbor wild-type (WT) PI3K p110*α* (Supplementary Figure S1f). This is in line with a recent report that selective inhibition of p110*α* using A66 is only efficient in preventing phosphorylation of AKT in cells with activating mutations in p110*α*.^28^

These results were unexpected but led us to conclude that PIK-75 sensitizes cancer cells to TRAIL-induced apoptosis either independently of p110*α* or by inhibiting p110*α* and (an) additional kinase(s). We therefore used PIK-75 in an *in vitro* screen testing its capability to inhibit a panel of 451 kinases (80% of the kinome). This revealed that, in addition to p110*α*, PIK-75 potently inhibited 27 other kinases when used at 200 nM (Figure 1e), a concentration at which it effectively sensitizes cancer cells to TRAIL. In conclusion, we established that PIK-75 potently breaks TRAIL resistance, but its p110*α*-inhibitory activity is either not responsible or alone not sufficient to sensitize cancer cells to TRAIL.

### CDK9 is the PIK-75-target responsible for TRAIL sensitization

**Figure 2:**
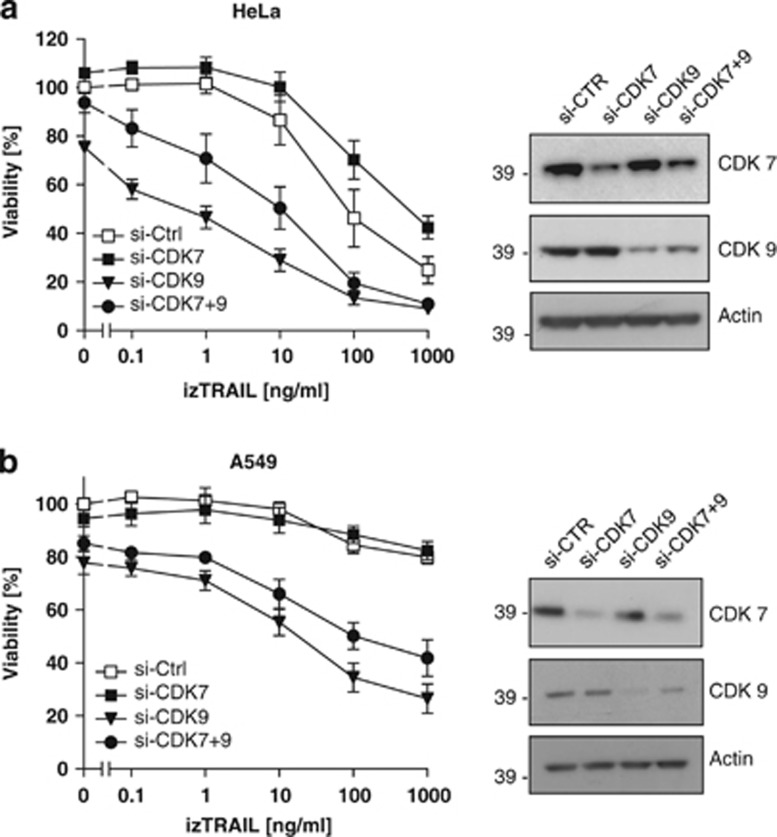
CDK9 is the PIK-75-target that is responsible for TRAIL sensitization. HeLa (**a**) or A549 cells (**b**) were transiently transfected with the indicated siRNAs for 48 h and subsequently stimulated with izTRAIL at different concentrations. Cell viability was determined 24 h later. Representative western blots of knockdown efficiency are shown. All values are means±S.E.M. of three independent experiments

To evaluate which of the 27 kinases inhibited, or which combination thereof, was responsible for PIK-75-mediated sensitization to TRAIL-induced apoptosis, we screened all 27 kinases identified in the *in vitro* screen by siRNA knockdown for sensitization to TRAIL (Supplementary Figure S2a). Knockdown of 26 of these kinases did not affect sensitivity to TRAIL. Silencing of cyclin-dependent kinase 9 (CDK9), however, potently sensitized HeLa and A549 cells to TRAIL-induced apoptosis (Figures 2a and b). CDK9 is a member of the family of CDKs, which are mainly known for their function in cell cycle regulation.^29^ Recently, it was shown that a subset of CDKs, namely CDK7 and CDK9 regulate transcription.^30, 31^ Our screen revealed that PIK-75 also inhibits CDK7. However, a role of CDK7 in mediating TRAIL resistance could be excluded, as CDK7 knockdown did not sensitize to TRAIL-induced apoptosis (Figures 2a and b). Moreover, a contributing role of the most prominent members of the cell cycle-regulating CDKs, CDK1, 2, 4 and 6 could also be excluded by knockdown experiments (Supplementary Figures S2b and c).

### CDK9 inhibition by SNS-032 potently sensitizes to TRAIL-induced apoptosis

**Figure 3:**
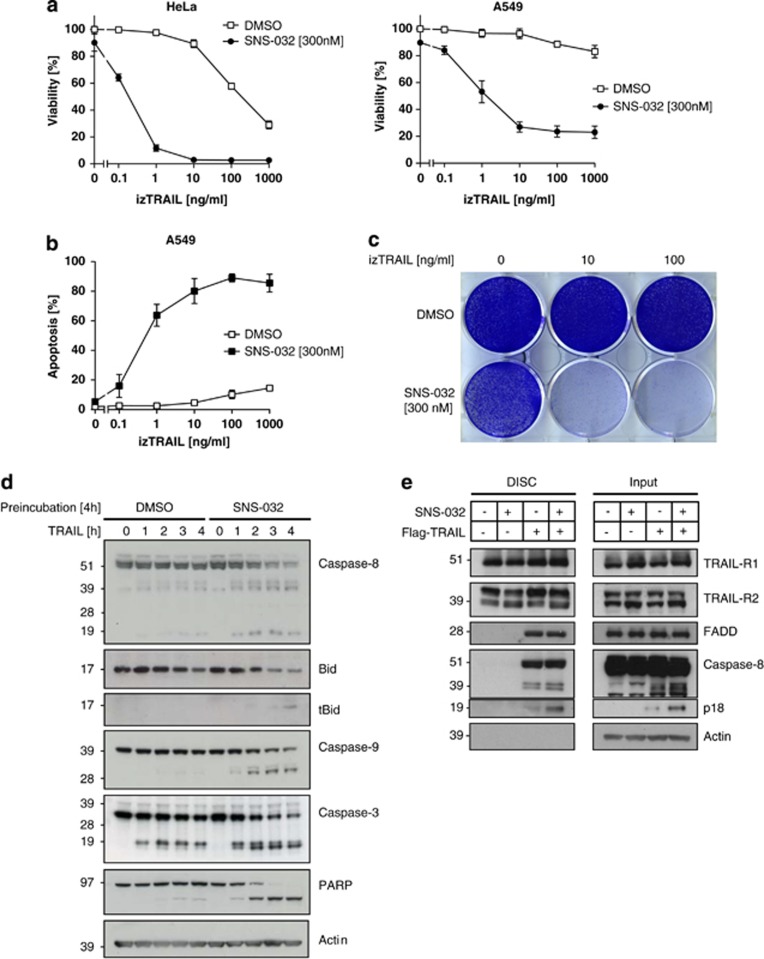
CDK9 inhibition by SNS-032 potently synergizes with TRAIL to kill cancer cells. (**a**) HeLa and A549 cells were preincubated with DMSO or SNS-032 (300 nM) for 1 h and subsequently stimulated with izTRAIL at the concentrations indicated. Cell viability was determined after 24 h. (**b**) A549 cells were preincubated with DMSO or SNS-032 (300 nM) for 1 h and subsequently stimulated with indicated concentrations of izTRAIL. Apoptosis was determined after 24 h. (**c**) A549 cells were treated with DMSO or SNS-032 (300 nM) for 1 h and subsequently stimulated for 24 h with izTRAIL (10 or 100 ng/ml). Long-term survival was visualized after 7 days by crystal violet staining. One of two independent experiments is shown. (**d**) A549 cells were preincubated with DMSO or SNS-032 (300 nM) for 4 h and subsequently stimulated with izTRAIL (100 ng/ml) for the indicated times. Cells were lysed and subjected to western blotting. One representative of two independent experiments is shown. (**e**) A549 cells were preincubated with SNS-032 (300 nM) for 12 h, stimulated with Flag-TRAIL (1 μg/ml) for 1 h and subsequently the TRAIL–DISC was immunoprecipitated via M2-coupled beads and analyzed by western blotting. One representative of two independent experiments is shown. All other values are means±S.E.M. of three independent experiments

Several CDK inhibitors targeting different subsets of CDKs are currently evaluated in clinical trials.^32^ Among them, SNS-032 (BMS-387032) appears to be the most selective CDK9 inhibitor. It inhibits CDK2, CDK7 and CDK9 selectively over other CDKs and kinases, but its inhibitory capacity is about 10-fold selective for CDK9 (IC50=4 nM) over CDK2 (IC50=38 nM) and 15-fold over CDK7 (IC50=62 nM).^33^ CDK9, in a complex with its partner Cyclin-T/K, constitutes the positive transcription elongation factor b (P-TEFb) that promotes transcriptional elongation by phosphorylation of substrates.^34, 35^ The most important substrate of P-TEFb is the carboxy-terminal domain of RNA-polymerase II (RNA-Pol II), which is phosphorylated by CDK9 at Ser-2. Analysis of Ser-2 phosphorylation of RNA-Pol II showed that PIK-75 and SNS-032 exerted similar inhibitory activity towards CDK9 (Supplementary Figure S3a). We next evaluated a novel combinatorial therapy consisting of the clinically used CDK9 inhibitor SNS-032 and TRAIL. Indeed, SNS-032 markedly sensitized HeLa and A549 cells to TRAIL-induced cell death (Figure 3a). Sensitized cells died apoptotically (Figure 3b) and this cell death was prevented by the caspase-inhibitor zVAD (Supplementary Figure S3b). Finally, SNS-032 in combination with TRAIL almost completely abrogated clonogenic survival of A549 cells (Figure 3c). These data demonstrate that cancer cell lines can be strongly sensitized to TRAIL-induced apoptosis via CDK9 inhibition using SNS-032, a small molecule inhibitor that is already undergoing clinical testing.

In line with these findings, cancer cells treated with TRAIL in the presence of SNS-032 showed a drastic increase in the cleavage of caspase-8, Bid, caspase-9, -3 and poly ADP ribose polymerase (PARP) (Figure 3d and Supplementary Figure S3c). Moreover, cells in which CDK9 was silenced using siRNA also showed increased activation of the apoptotic caspase cascade (Supplementary Figure S3d). As expected from this finding, DISC analysis upon CDK9 inhibition using SNS-032 (Figure 3e) or upon CDK9 knockdown (Supplementary Figure S3e) revealed that caspase-8 cleavage generating the p18 fragment was enhanced upon CDK9 inhibition or suppression at the DISC (Figure 3e, Supplementary Figure S3e). Thus, CDK9 inhibition facilitates initiation of the caspase cascade at the DISC as part of its sensitization mechanism.

### CDK9 mediates TRAIL resistance by promoting concomitant transcription of cFlip and Mcl-1

**Figure 4:**
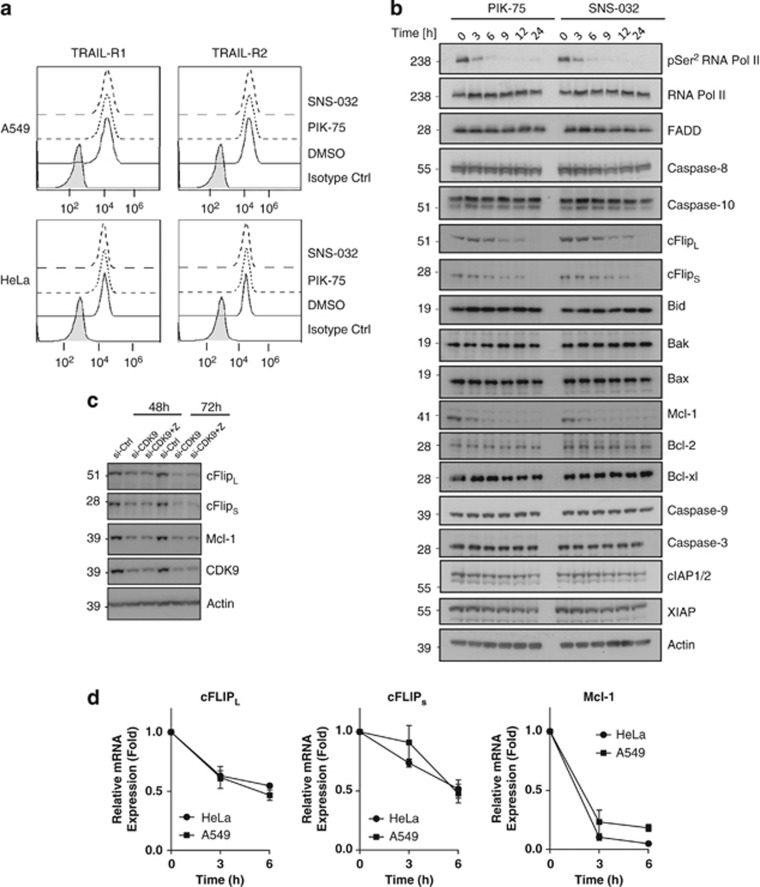
CDK9 mediates TRAIL resistance by promoting concomitant transcription of cFlip and Mcl-1. (**a**) A549 or HeLa cells were incubated with SNS-032 (300 nM) or PIK-75 (100 nM) for 6 h and subsequently stained for surface expression of TRAIL-R1 and TRAIL-R2. One representative of two independent experiments is shown. (**b**) A549 cells were treated with PIK-75 (100 nM) or SNS-032 (300 nM) for the indicated times. Cells were lysed and subjected to western blotting. One representative of two independent experiments is shown. (**c**) HeLa cells were subjected to the indicated knockdowns for 48 or 72 h. zVAD was added at 20 *μ*M 24 h after transfection where indicated. Cells were lysed and subjected to western blotting. One representative of two independent experiments is shown. (**d**) A549 and HeLa cells were incubated with SNS-032 (300 nM) for different times. cFlip_L_, cFlip_S_ and Mcl-1 mRNA expression was quantified by RT-PCR. Values are means±S.E.M. of three independent experiments. Z, zVAD

Having established that CDK9 inhibition efficiently sensitizes cancer cell lines to TRAIL-induced apoptosis, we next addressed which molecular changes are responsible for this effect. Upregulation of TRAIL-R1 and/or TRAIL-R2 often correlates with, and sometimes also contributes to, TRAIL apoptosis sensitization.^36^ However, treatment of HeLa or A549 cells with PIK-75 or SNS-032 did not alter TRAIL-R1/R2 surface expression (Figure 4a), in line with similar recruitment of TRAIL-R1/2 in the DISC analysis (Figure 3e). Consequently, TRAIL sensitization by CDK9 inhibition is likely to require changes in intracellular modulators of the TRAIL apoptosis pathway that should enhance DISC activity and possibly additional downstream steps in the pathway. We, therefore, next investigated whether known components of the TRAIL–DISC and the downstream apoptosis pathway it activates are regulated by PIK-75 or SNS-032 treatment. Whereas the majority of the DISC components and downstream pro- and anti-apoptotic proteins remained unchanged, cFlip and Mcl-1 protein levels were rapidly suppressed by pharmacological CDK9 inhibition by SNS-032 or PIK-75 (Figure 4b and Supplementary Figure S4a). Because siRNA-mediated suppression of CDK9, performed in the presence or absence of pan-caspase inhibition to exclude a possible impact of CDK9-silencing-induced apoptosis, also resulted in downregulation of cFlip and Mcl-1, we can conclude that CDK9 is required to maintain high expression of these anti-apoptotic proteins in cancer cells (Figure 4c).

CDK9 is known for its role in transcriptional elongation, suggesting that the observed downregulation of cFlip and Mcl-1 protein levels could be caused by suppression of their transcripts. In line with this hypothesis, SNS-032 treatment rapidly decreased the amount of mRNA for cFlip and Mcl-1 (Figure 4d). The effect was a consequence of direct inhibition of transcription, because co-treatment with SNS-032 and the transcriptional inhibitor actinomycin D^37^ did not further reduce mRNA levels (Supplementary Figure S4b). Moreover, preincubation with the translational inhibitor cycloheximide before SNS-032 treatment did not inhibit SNS-032-mediated mRNA suppression (Supplementary Figure S4b). Co-incubation with actinomycin D and cycloheximide induced a steady-state level of mRNA. Additional treatment with SNS-032 did not reduce Mcl-1 mRNA, showing that SNS-032 does not induce degradation of mRNA. Next, we analyzed cFlip and Mcl-1 mRNA upon CDK9 knockdown. In slight contrast to CDK9 inhibition using SNS-032, prolonged silencing of CDK9 using siRNA also strongly affected mRNA levels of housekeeping genes. Therefore, we normalized mRNA amounts to cell numbers used for RNA extraction. The amplification of cFlip and Mcl-1 transcripts by real-time PCR (RT-PCR) required a higher cycle threshold, demonstrating that their transcripts are indeed suppressed when normalized to the cell number (Supplementary Figure S4c). We conclude that SNS-032-induced suppression of cFlip and Mcl-1 is mediated by direct inhibition of global transcription that will preferentially affect expression levels of short-lived proteins such as cFlip and Mcl-1.

### Concomitant downregulation of cFlip and Mcl-1 is sufficient and required for CDK9 inhibition-induced TRAIL sensitization

**Figure 5:**
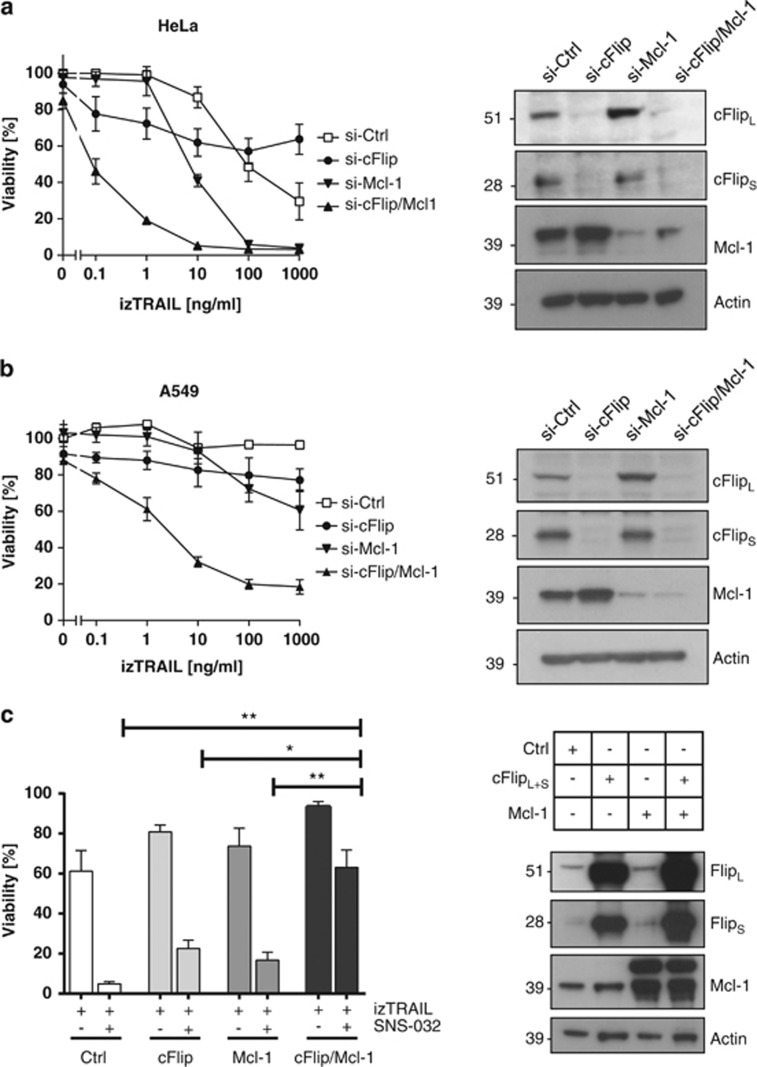
Concomitant downregulation of cFlip and Mcl-1 is required and sufficient for CDK9 inhibition-induced TRAIL sensitization. HeLa (**a**) and A549 cells (**b**) were transfected with siRNA-targeting cFlip and/or Mcl-1 for 48 h and subsequently stimulated with izTRAIL at the indicated concentrations. Cell viability was determined after 24 h. (**c**) HeLa cells were transfected with expression plasmids for cFlip and/or Mcl-1 or empty vector control. Twenty four hours later, cells were stimulated with izTRAIL (10 ng/ml) for 24 h and cell viability was determined. All values are means±S.E.M. of three independent experiments. Representative western blots are shown. *P<0.05; **P<0.01; Student's *t*-test

To evaluate whether concomitant suppression of cFlip and Mcl-1 was sufficient for CDK9 inhibition-mediated TRAIL sensitization, we silenced cFlip and/or Mcl-1 in HeLa and A549 cells. Hela cells were sensitized to die by Mcl-1 knockdown alone only when high concentrations of TRAIL were used. Knockdown of cFlip, in turn, sensitized at lower TRAIL concentrations, whereas at higher TRAIL concentrations HeLa cells were rendered more resistant by cFlip knockdown (Figure 5a). The latter may be attributable to the interesting observation that knockdown of cFlip brought about the upregulation of Mcl-1. In A549 cells, silencing of neither cFlip nor Mcl-1 alone was sufficient to sensitize to TRAIL-induced apoptosis (Figure 5b). Combined knockdown of both components, however, resulted in a striking synergistic sensitization rendering both, HeLa and A549 cells, highly susceptible to TRAIL-induced apoptosis (Figures 5a and b). Thus, combined downregulation of cFlip and Mcl-1 is sufficient to break TRAIL resistance.

To further investigate the interesting observation that silencing of either cFlip or Mcl-1 resulted in the inverse upregulation of the respective other protein, we also analyzed transcripts of cFlip and Mcl-1 upon knockdown. Silencing of cFlip, Mcl-1 or the combination thereof resulted in comparable and efficient suppression of the respectively targeted transcripts (Supplementary Figure S5a). Interestingly, the inverse upregulation we observed on the protein level was also apparent on the transcriptional level (Supplementary Figure S5a), suggesting that this phenomenon is, at least partially, regulated on the transcriptional level.

To test whether cFlip and/or Mcl-1 were responsible for the block of TRAIL-induced apoptosis that is specifically removed by CDK9 inhibition, we overexpressed cFlip and/or Mcl-1 in HeLa cells before treatment with SNS-032 and TRAIL. Transfection was highly efficient (Supplementary Figure S5b) and nontoxic to the cells (Supplementary Figure S5c). Overexpression of cFlip or Mcl-1 alone rendered these cells slightly more TRAIL resistant but could only marginally inhibit SNS-032-mediated sensitization (Figure 5c). Combined overexpression, however, rendered HeLa cells almost completely resistant to TRAIL-induced apoptosis and prevented SNS-032-mediated sensitization (Figure 5c). Thus, SNS-032 sensitizes cancer cell lines to TRAIL-induced apoptosis by concomitant suppression of cFlip and Mcl-1.

We next investigated whether CDK9 inhibition-induced TRAIL sensitization requires activation of the mitochondrial pathway. To do so, we used the isogenic HCT-116 colon carcinoma cell lines in which Bax and Bak are either both expressed (parental HCT-116 WT cells) or both genetically deleted (BAX/BAK-deficient HCT-116 cells). HCT-116 WT cells were partially TRAIL sensitive but profoundly sensitized by co-treatment with SNS-032 (Supplementary Figure S5d). Their Bax/Bak-deficient counterparts, however, were completely resistant to SNS-032-mediated TRAIL sensitization. Thus, TRAIL sensitization mediated by CDK9 inhibition uses a type-II apoptosis pathway that requires both, effective DISC-mediated caspase-8 activation with consequent Bid cleavage, enabled by cFlip downregulation, and efficient triggering of the mitochondrial apoptosis pathway by cleaved Bid, enabled by Mcl-1 downregulation.

### Combined CDK9 inhibition and TRAIL selectively kills NSCLC cell lines but not primary human hepatocytes within a therapeutic window

**Figure 6:**
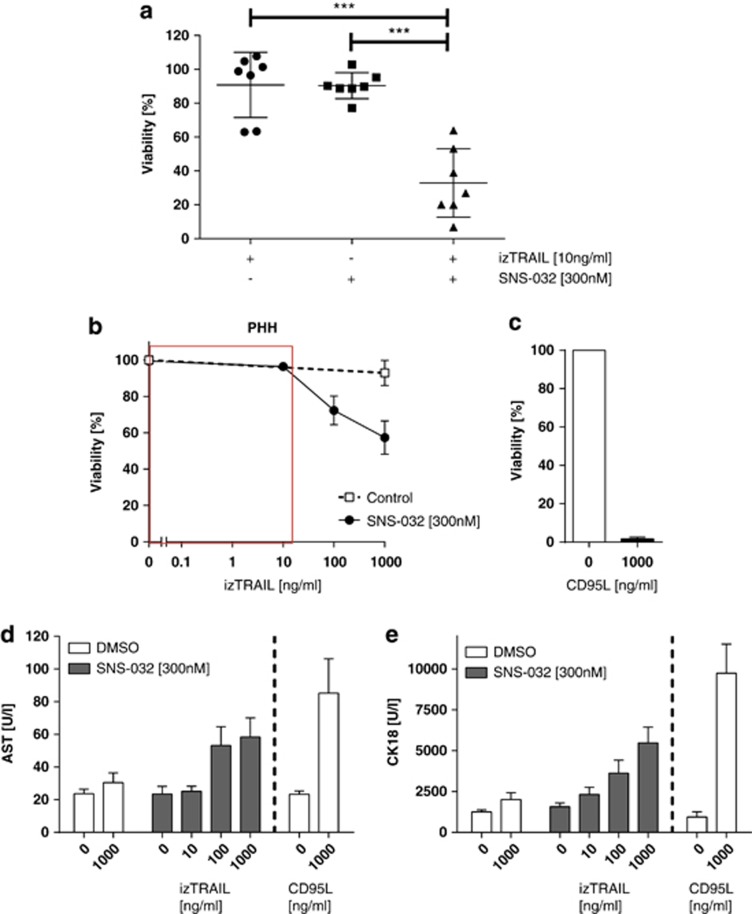
Combination of TRAIL and CDK9 inhibition selectively kills NSCLC cell lines but not PHH within a therapeutic window. (**a**) Seven NSCLC cell lines were preincubated with SNS-032 (300 nM) for 1 h and subsequently stimulated with izTRAIL (10 ng/ml). Cell viability was quantified after 24 h. Values are means of ±S.D. Individual dots represent means of three independent experiments of one cell line. (**b**) On day 4 of culture, PHH of three different donors were preincubated with DMSO or SNS-032 (300 nM) for 1 h and stimulated with izTRAIL at the indicated concentrations. Cell viability was analyzed after 24 h. (**c**) PHH were treated with CD95L (1 *μ*g/ml) as positive control. Supernatants of treated PHH were used to determine levels of AST (**d**) and caspase-cleaved cytokeratin 18 (**e**). Values are means of three independent experiments±S.E.M. ***P<0.001; Student's *t*-test

On all cancer cell lines tested, including primarily TRAIL-resistant A549 cells, already low concentrations of TRAIL (1–10 ng/ml) in the presence of SNS-032 (300 nM) were sufficient to reach maximum efficiency in killing these cells. To investigate whether this was a coincidence or may be applicable more broadly, we extended our study to an established panel of NSCLC cell lines.^38^ This panel includes cells that are mutated in *KRAS* and/or *p53* (Supplementary Figure S6a). The majority of the cell lines were TRAIL resistant, resembling TRAIL sensitivity of primary cancer cells (Figure 6a and Supplementary Figure S6b). However, all cell lines tested were potently sensitized to 10 ng/ml of TRAIL by co-treatment with SNS-032 at 300 nM, irrespective of their oncogenic mutations (Figure 6a and Supplementary Figure S6b). Thus, SNS-032/TRAIL co-treatment enables efficient killing in a broad range of cancer cell lines, irrespective of their *p53*-status.

Considering the remarkable sensitization observed with combination of TRAIL and SNS-032, we next tested the cancer selectiveness of this new combination. Hepatotoxicity is a major concern for the clinical application of novel cancer therapeutics and special care should be taken in the development of therapies containing TNF superfamily members.^3^ We therefore next assessed the effect of TRAIL and/or SNS-032 treatment on primary human hepatocytes (PHH). In line with our previous results,^39^ the recombinant form of TRAIL used in our study (izTRAIL) did not reduce viability of PHH (Figure 6b). In contrast, PHH were readily killed by recombinant CD95L that served as a control (Figure 6c). Treatment of PHH with SNS-032 at 300 nM in combination with TRAIL used at different concentrations revealed that at high concentrations of TRAIL (100 ng/ml and 1000 ng/ml) hepatocytes died when co-treated with SNS-032 (Figure 6b). However, co-treatment with SNS-032 at 300 nM and TRAIL at 10 ng/ml, the concentrations at which these drugs were highly efficient at killing cancer cells when combined, did not affect viability of hepatocytes. The same nontoxic window was confirmed for the levels of aspartate transaminase (AST), which is released when liver cells are damaged (Figure 6d), and the levels of caspase-cleaved cytokeratin 18 (Figure 6e). Therefore, our novel therapeutic combination can be applied within a considerable therapeutic window. At the same time, toxicity would be expected at higher levels of TRAIL.

### TRAIL combined with CDK9 inhibition eradicates established orthotopic lung tumors

**Figure 7:**
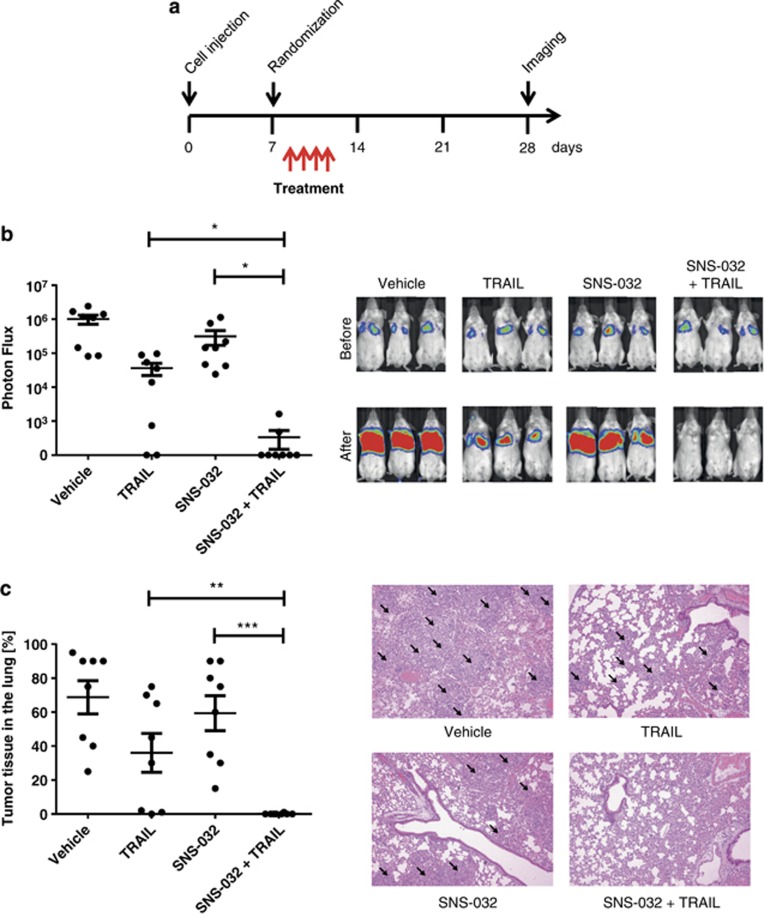
SNS-032 and TRAIL co-treatment eradicates established lung tumors *in vivo*. (**a**) Experimental treatment schedule is shown. (**b**) In week three after treatment tumor burden was quantified by bioluminescence imaging (Photon Flux). Values are means ±S.E.M. Dots represent individual mice (*n*=8 per group). Three representative mice from each group are shown. (**c**) Paraffin sections of lungs from all mice were stained with H&E and subjected to microscopical analysis quantifying the percentage of total lung area occupied by tumour tissue. Values are means ±S.E.M. Dots represent lungs from individual mice, (*n*=8 per group). Representative histological images are shown (arrows indicate tumor tissue). *P<0.05; **P<0.01, ***P<0.001; Student's *t*-test

Having established an applicable therapeutic window for our newly identified combination of TRAIL with SNS-032 *in vitro*, we next assessed this combination's potency in an orthotopic model of lung cancer *in vivo*. To this end, we induced lung tumors via tail vein injection of A549 cells stably expressing luciferase (A549-luc). After 7 days, mice were randomized to create treatment groups of mice with comparable tumor burden in each group (Supplementary Figure S7). Subsequently, a 4-day treatment regime was started with either vehicle, TRAIL, SNS-032 or the combination of SNS-032 and TRAIL (Figure 7a). Whereas TRAIL treatment alone had a slight growth inhibitory effect, and SNS-032 only marginally affected lung tumor burden, combined treatment with TRAIL and SNS-032 induced a drastic antitumor effect. TRAIL/SNS-032 treatment completely eradicated established lung tumors in most mice, as determined by *in vivo* bioluminescence imaging (Figure 7b) and subsequent histopathological inspection of lung sections (Figure 7c). Strikingly, and in line with the bioluminescence data, seven out of eight mice that had received TRAIL combined with SNS-032 were histologically tumor free after a 4-day treatment cycle.

## Discussion

We found that the supposedly p110*α*-specific inhibitor PIK-75 potently sensitizes to TRAIL-induced apoptosis. Surprisingly, however, PI3K inhibition was not responsible for this effect. A kinome-wide screen revealed that PIK-75 strongly inhibits 27 kinases in addition to p110*α*. Off-target activity is a common feature among kinase inhibitors, as most inhibitors are ATP-competitive compounds and the ATP-binding pocket is highly conserved among the human kinome.^40, 41^ We show that PIK-75 exerts off-target effects toward CDK7 and CDK9. This is in line with a recent report on the effects of PIK-75 on acute myeloid leukemia.^42^ Moreover, we demonstrate that PIK-75's activity to sensitize cancer cells to TRAIL-induced apoptosis is exclusively due to inhibition of CDK9. CDKs are mainly known for their regulatory role in cell cycle, and development of CDK inhibitors for cancer therapy is aimed at suppressing exacerbated cell cycle progression.^43^ Recently, a subset of CDKs, namely CDK7 and CDK9, has been implicated in regulating transcription.^30, 31^ CDK9 inhibition has been shown to block transcriptional elongation, thereby suppressing expression of short-lived proteins such as Mcl-1 that can result in induction of apoptosis in cancer cells.^30^ This finding has paved the way for targeting transcriptional CDKs in addition to cell cycle-regulating CDKs in cancer therapy. Here we provide evidence that selective inhibition of CDK9 achieves an exceptionally potent sensitization to TRAIL-induced apoptosis. Interestingly, the pan-CDK inhibitors Flavopiridol^44, 45, 46^ and Roscovitine (Seliciclib)^47, 48, 49^ have previously been shown to synergize with TRAIL. However, so far, it remained unclear which CDK, inhibited by these pan-CDK inhibitors, was responsible for these effects. When combining our result with the fact that Flavopiridol and Roscovitine also inhibit CDK9, it appears reasonable to assume that their previously described TRAIL-sensitizing capacity is likely owed to their CDK9-inhibitory capacity.

Inhibition of certain CDKs can potentially cause toxicity, and CDK1 inhibition is currently thought to be most problematic in this respect.^50^ To avoid potential dose-limiting toxicity, we devised a novel combinatorial therapy consisting of TRAIL and SNS-032, an inhibitor targeting CDK9 preferentially over cell cycle CDKs.^33^ Importantly, the safety of SNS-032 was already confirmed in clinical trials^51, 52^ and SNS-032 has been shown to be more potent in inhibiting transcription than Flavopiridol and Roscovitine.^53^ The fact that CDK9 inhibition was found to be nontoxic in clinical trials implies that normal cells have possibly developed coping mechanisms that might not be present in transformed cells. In line with this notion, our results show that CDK9 inhibition in combination with TRAIL can selectively kill tumor cells, but not PHH within a significant therapeutic window. Of note, the concentration at which SNS-032 effectively sensitizes cancer cells to TRAIL-induced apoptosis, 300 nM, is commonly reached and sustained in the plasma of patients.^51^

Investigating the underlying mechanism of how CDK9 inhibition sensitizes to TRAIL-induced apoptosis revealed that Mcl-1 downregulation is required, but not sufficient, for TRAIL sensitization. In addition, CDK9 inhibition-induced suppression of another short-lived protein, cFlip, was required to achieve potent TRAIL sensitization. Hence, the synergistic effect of CDK9 inhibition and TRAIL is due to a dual mechanism: downregulation of cFlip enables caspase-8 activation at the DISC and downregulation of Mcl-1 facilitates activation of the mitochondrial apoptosis pathway for enhanced caspase-9 and, ultimately, caspase-3 activation. As a consequence, the combination of TRAIL and CDK9 inhibition is exquisitely powerful in killing tumor cells with a cFlip-imposed block to initiator caspase activation at the DISC and an Mcl-1-imposed block to activation of the mitochondrial apoptosis pathway.

Chemotherapy mostly induces apoptosis by induction of DNA damage that is sensed by p53.^54^ However, impairment of functional p53, either by mutation or loss of expression, is frequently detected in cancer. Therefore, therapies that function independently of *p53*-status are likely to be more effective than chemotherapy. Importantly, we determined that CDK9 inhibition sensitizes cancer cells to TRAIL irrespective of their *p53*-status, thereby providing a therapeutic option also for cancers with mutated *p53* in which conventional chemotherapy is largely ineffective. Moreover, the high efficacy of the newly devised treatment combination was also apparent *in vivo*. In an orthotopic lung cancer xenograft model, the combination of SNS-032 with TRAIL eradicated established lung tumors after a 4-day treatment cycle. This striking result provides further support for the high therapeutic potential of combinations of TRAIL-R agonists with CDK9 inhibitors.

Recent reports on first clinical trials with TRAIL and other TRAIL-R agonists showed, on the one hand, that these biotherapeutics were well tolerated but, on the other, that the clinical activity they exerted, even when combined with standard chemotherapy, was rather limited.^6^ Cancer cell resistance to TRAIL-induced apoptosis is likely to be a significant factor in this outcome, indicating that a TRAIL-comprising therapy will only be effective when a potent TRAIL sensitizer is applied in combination with a TRAIL-R agonist. Based on our results, we propose CDK9 inhibition as an effective means to overcome TRAIL resistance in a cancer-selective manner.

## Materials and Methods

### Reagents

Antibodies: *α*-RNA-Pol II, *α*-pSer2 and *α*-pSer5 were purchased from Covance (Princeton, NJ, USA); *α*-Caspase-3 and *α*-cIAP from R&D Systems (Abingdon, UK); *α*-cFlip (NF6) and *α*-Caspase-8 (C15) are available from Enzo (Exeter, UK); *α*-PARP was purchased from BD Biosciences (Oxford, UK); *α*-FADD was purchased from BD Biosciences (IgG1) or Santa Cruz (Heidelberg, Germany) (rabbit). *α*-Caspase-10 and *α*-Caspase-9 from MBL (Woburn, MA, USA); *α*-*β*-Actin from Sigma (Gillingham, UK) and *α*-DNA-PK, *α*-p110α, *α*-p110β, *α*-Bak, *α*-Bax, *α*-Mcl-1, *α*-Bcl-2, *α*-Bcl-xL, *α*-XIAP, *α*-CDK1, *α*-CDK2, *α*-CDK4, *α*-CDK6, *α*-CDK7, *α*-CDK9, *α*-AKT and *α*-pAKT(Ser473) from Cell Signaling (Danvers, MA, USA); *α*-Bid was obtained from or Cell Signaling (rabbit) or R&D Systems (goat). HS101 and HS201 were used for surface staining of TRAIL-R1/–R2 and are available from Enzo (Exeter, UK). Recombinant TRAIL was used as an isoleucine zipper-tagged version of the extracellular domain of human TRAIL (izTRAIL) as described previously.^39^ PIK-75, TGX-221 AS-252424, IC-87144, A66, BEZ-235, GDC-0941 and SNS-032 were purchased from Selleck Chemicals (Houston, TX, USA); actinomycin D from Merck Millipore (Darmstadt, Germany); cycloheximide and crystal violet from Sigma, z-VAD(OMe)-FMK from Abcam (Cambridge, UK) and D-Luciferin from Caliper Life Science (Waltham, MA, USA).

### Cell lines

The human lung adenocarcinoma panel (H460, H522, H322, H441, Calu-1 and H23) was kindly provided by J Downward and cultured in RPMI supplemented with 10% FCS. A549-luc cells were purchased from Caliper Life Science and cultured in RPMI supplemented with 10% FCS. HeLa cells were cultured in DMEM supplemented with 5% FCS. HCT-116 WT and HCT-116 ^Bax-/-Bak-/-^ were kindly provided by B Vogelstein and R Youle and were cultured in DMEM supplemented with 10% FCS. PHHs were purchased from Gibco/Invitrogen (Paisley, UK) and cultured according to the manufacturer's instructions.

### RNA interference

siRNA pools (ON-TARGET plus) containing four different siRNA sequences targeting each gene of interest were purchased from Dharmacon/Thermo Scientific (Loughborough, UK). Cells were transfected using Dharmafect reagent according to the manufacturer's instructions. Cells were used for further analysis at 48 or 72 h after transfection. Knockdown efficiency was assessed by western blot in parallel.

### Cell viability and cell death assays

Cell viability was determined using the Cell Titer Glo assay (Promega, Southampton, UK) according to the manufacturer's instructions. As a direct measurement of apoptotic cell death, DNA fragmentation was quantified as described before.^55^ To analyze long-term survival (clonogenic assay), cells were seeded into six-well plates. The next day, cells were preincubated with DMSO, PIK-75 or SNS-032 for 1 h before izTRAIL was added. After 24 h, dead cells were washed away and surviving cells were cultured for additional 6 days in fresh medium without any treatment. After 7 days, cells were washed twice with PBS, fixed with 10% formaldehyde in PBS for 30 min at room temperature and stained with crystal violet (1% in 50% ethanol).

### Western blot analysis

Cells were treated as indicated and then lysed in lysis buffer (30 mM Tris-HCl; pH 7.4, 150 mM NaCl, 2 mM EDTA, 2 mM KCl, 10% glycerol, 1% Triton X-100 and 1 × complete protease-inhibitor cocktail (Roche, Burgess Hill, UK)). Proteins were separated by SDS-PAGE (NuPAGE) and analyzed by western blotting. Membranes were stripped with 50 mM glycine (pH 2.3) before reprobing with other antibodies.

### DISC analysis

We performed ligand affinity precipitations using Flag-tagged TRAIL in combination with M2 beads (Sigma). Cells were incubated for 1 h at 37 °C in the presence or absence of 1 *μ*g/ml Flag-TRAIL. For the precipitation of the non-stimulated receptors, Flag-TRAIL was added to the lysates prepared from non-stimulated cells. Precipitates were prepared as described previously.^56^

### TRAIL-R surface staining

Cells were detached using Accutase (Sigma) and counted. Cells (2 × 10^5^) were incubated with 10 *μ*g/ml anti-TRAIL-R1 (HS101) or anti-TRAIL-R2 (HS201) or IgG1 isotype control antibody in 2% BSA in 100 *μ*l PBS (BSA/PBS) for 30 min on ice. Cells were washed twice with ice-cold BSA/PBS before incubation with secondary goat–anti-mouse-APC (BioLegend, London, UK) at a dilution of 1:200 in BSA/PBS for 20 min on ice. Cells were washed three times in ice-cold BSA/PBS and surface expression was assessed by flow cytometry.

### Overexpression of cFlip and Mcl-1

HeLa cells were transfected with control, PEGZ-cFlip, pEF 3xFLAG-hMcl-1 or both using Lipofectamine LTX (Invitrogen, Paisley, UK) according to the manufacturer's instructions. Cells were left untreated for 24 h before any treatment to ensure efficient expression of the respective protein. Efficient expression of the respective protein was controlled by SDS-PAGE and subsequent western blot. Furthermore, cells were transfected with a GFP-containing plasmid and transfection efficiency was quantified by flow cytometry.

### Determination of AST values

Supernatant (30 *μ*l) of treated PHHs was used to determine AST levels using a Reflovet Analyzer (Roche) and Reflotron GOT test strips according to the manufacturer's instructions.

### Caspase-cleaved CK 18-ELISA

Supernatant (50 *μ*l) of treated PHHs was used in the M30 Apoptosense ELISA (Peviva, Bromma, Sweden) according to the manufacturer's instructions.

### High-Throughput kinase selectivity profiling (Kinomescan)

High-throughput kinase selectivity profiling assay (Kinomescan, DiscoveRx, Fremont, CA, USA) was used to determine the promiscuity of PIK-75 as a kinase inhibitor. The capacity of PIK-75 to bind to a panel of 451 human kinases was determined by analyzing the binding interaction (%) compared with DMSO (=100%). We chose to use PIK-75 at 200 nM in this screen because this was twice the concentration of this agent required to sensitize cancer cells to TRAIL. Hits were visualized using the TREEspot visualization tool provided by DiscoveRx. Kinases were considered hits if their activity was inhibited by >90% leaving <10% remaining activity.

### RNA analysis by RT-PCR

RNA was extracted using the RNeasy Kit (Qiagen, Manchester, UK) and treated with the TURBO DNA-free Kit (Ambion, Paisley, UK) according to the manufacturer's instructions. cDNA was generated using the RevertAid H Minus Strand cDNA Synthesis Kit (Thermo Scientific, Loughborough, UK) and used in combination with the FastStart Universal ProbeLibrary Mastermix (Roche) for the RT-PCR. Quantification of gene products was performed using the Eppendorf Mastercycler. When fold changes are shown, the gene product was normalized to GAPDH as a housekeeping gene and were calculated using the method described by Pfaffl *et al.*^57^

### Primers for RT-PCR

Primers and probe combinations were determined using the Universal ProbeLibrary Design Center (Roche) and are as follows.

cFLIP(s) forward: 5′–TTGGAAATTGTTCCATGTGATT-3′

cFLIP(s) reverse: 5′-GCAACAAGAAAGGGCTAAACA-3′

cFLIP(s) Essay Universal ProbeLibrary Number: 34

cFLIP(l) forward: 5′-GCTCACCATCCCTGTACCTG-3′

cFLIP(l) reverse: 5′-CAGGAGTGGGCGTTTTCTT-3′

cFLIP(l) Essay Universal ProbeLibrary Number: 14

CDK9 forward: 5′-TTCGGGGAGGTGTTCAAG-3′

CDK9 reverse: 5′-ATCTCCCGCAAGGCTGTAAT-3′

CDK9 Essay Universal ProbeLibrary Number: 21

MCL-1 forward: 5′-AAGCCAATGGGCAGGTCT-3′

MCL-1 reverse: 5′-TGTCCAGTTTCCGAAGCAT-3′

MCL-1 Essay Universal ProbeLibrary Number: 49

GAPDH forward: 5′-AGCCACATCGCTCAGACAC-3′

GAPDH reverse: 5′-GCCCAATACGACCAAATCC-3′

GAPDH Essay Universal ProbeLibrary Number: 60

### Orthotopic lung cancer xenograft

Female Fox Chase SCID Beige Mice (6–12 week old; Charles River, Germany) were injected with 2 × 10^6^ A549-luc cells via the lateral tail vein. After 1 week, all mice were imaged for bioluminescence using the Ivis Spectrum (Caliper Life Science). Photons per second (Photon Flux) were quantified using the Ivis Spectrum software. Mice with established tumor burden were included in the study and randomized into the treatment groups (eight mice/group). Subsequently, mice were treated for 4 consecutive days with daily i.p. injections of 600 *μ*g SNS-032 (30 mg/kg) and/or 100 *μ*g izTRAIL or 200 *μ*l buffer as control. After 3 weeks, tumor burden was quantified by bioluminescence imaging. For preparation of lung tissue sections, mice were killed according to Guidance on Operation of Animals (Scientific Procedures) Act 1986. Lungs were removed, fixed in 10% formalin for 1 week and then transferred to 70% ethanol. Paraffin embedding, preparation of sections and H&E stainings were performed as part of a histological staining service at the National Heart & Lung Institute. H&E stainings were examined and quantified by an experienced pathologist (MAE-B) who was blinded to the study. Tumor burden was quantified as percentage of tumor tissue in the lung. SCID beige mice were maintained in individually ventilated cages, received autoclaved food, water and bedding according to the institutional guidelines under a UK Home Office project license. The required risk assessments were obtained for this study.

### Statistical analysis

Data were analyzed using GraphPad Prism 6 software (GraphPad Software). Statistical significance between groups was determined using Student's *t*-test. Significant *P*-values are denoted as *P<0.05; **P<0.01; ***P<0.001.

## Abbreviations

AST: aspartate transaminase
CDK: cyclin-dependent kinase
cFlip: cellular FLICE-like inhibitory protein
DD: death domain
DISC: death-inducing signaling complex
FADD: Fas-associated protein with death domain
IAP: inhibitor of apoptosis proteins
NSCLC: non-small cell lung cancer
PI3K: phosphoinositide-3 kinase
PHH: primary human hepatocytes
P-TEFb: positive transcription elongation factor b
RNA Pol II: RNA-polymerase II
TNF: tumor necrosis factor
TRAIL: tumor necrosis factor-related apoptosis-inducing ligand
WT: wild-type
XIAP: X-linked inhibitor of apoptosis

## Notes

HW is co-founder, scientific advisor and shareholder of Apogenix GmbH (Heidelberg, Germany), a company that develops apoptosis-based drugs. The remaining authors declare no conflict of interest.
