# Supplementary material for: Selective CDK9 inhibition overcomes TRAIL resistance by concomitant suppression of cFlip and Mcl-1
Source: Cell Death Differ. 2013 Dec 20;21(3):491–502. doi: 10.1038/cdd.2013.179 (PMC3921597; doi:10.1038/cdd.2013.179)
Supplement: Supplementary Information [file cdd2013179x1.pdf]

Supplementary Information

Selective CDK9 inhibition overcomes TRAIL resistance by concomitant suppression of cFlip and Mcl-1

Johannes Lemke[1,2], Silvia von Karstedt[1], Muad Abd El Hay[1], Annalisa Conti[1,3], Frederick Arce[4], Antonella Montinaro[1], Kerstin Papenfuss[1], Mona A. El-Bahrawy[5], Henning Walczak[1]

[1] Centre for Cell Death, Cancer and Inflammation, UCL, 72 Huntley Street, London WC1E 6DD, UK

[2] Clinic of General and Visceral Surgery, University of Ulm, Albert-Einstein-Allee 23, 89081 Ulm, Germany

[3] Department of Experimental Oncology and Molecular Medicine, Fondazione IRCCS Istituto Nazionale dei Tumori, 20133 Milan, Italy

[4] Cancer Immunology Unit, UCL, 72 Huntley Street, London WC1E 6DD, UK

[5] Department of Histopathology, Imperial College London, Du Cane Road, London W12 0NN, UK

Corresponding author: Henning Walczak, E-mail: h.walczak@ucl.ac.uk, phone: +44 207 679 46471; FAX: +44 207 679 6925

Running Title: CDK9 inhibition overcomes TRAIL resistance

This file contains Figures S1 – S7 with legends and supplementary literature

A

IC50 [nM]	p110α	p110β	p110γ	p110δ	Ref.
PIK-75	6	1300	76	510	[1]
TGX-221	5000	5	>10000	100	[2]
AS-252424	940	>10.000	30	>10.000	[3]
IC-87114	>10.000	>10.000	>10.000	130	[1]

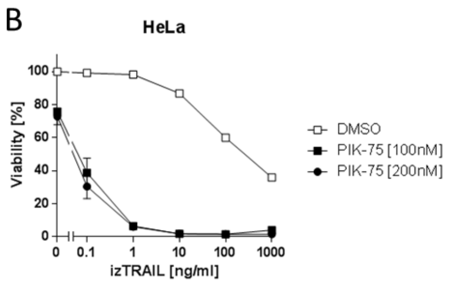

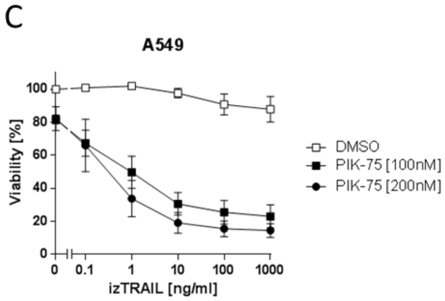

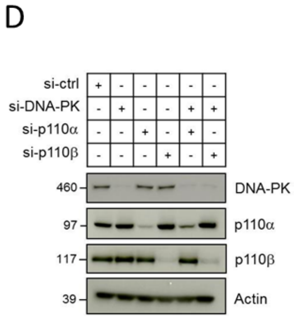

E

IC50 [nM]	p110α	p110β	p110γ	p110δ	Ref.
GDC-0941	3	33	75	3	[4]
A66	32	>10.000	>1250	3480	[5]
BEZ-235	4	75	5	7	[6]

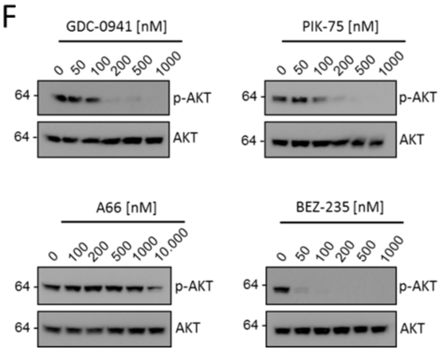

Figure S1 (a) IC50 values for PI3K isoform-specific inhibitors used in Figure 1a are shown. HeLa (b) or A549 (c) cells were pre-incubated with the indicated concentration of PIK-75 for 1h and subsequently stimulated with the indicated concentration of izTRAIL. Cell viability was quantified after 24h. Values represent means ± SEM of three independent experiments. (d) Westernblot demonstrating the knockdown efficiency for Figure 1c is shown. (e) IC50 values for PI3K inhibitors used in Figure 1d are shown. (f) HeLa cells were stimulated with different PI3K inhibitors at the indicated concentrations for 1h. Cells were lysed and subjected to western blotting. One representative of two independent experiments is shown.

A

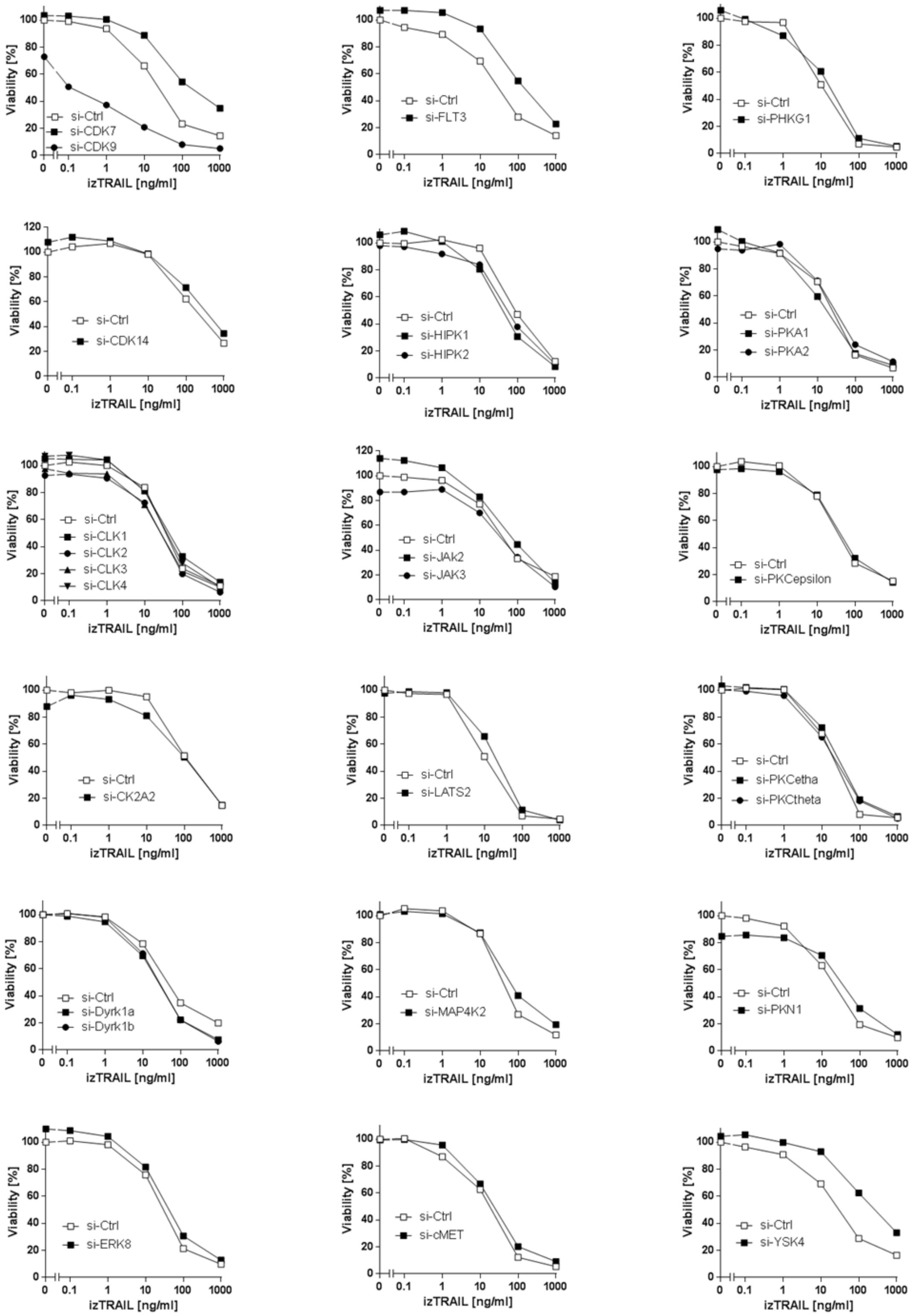

Viability [%]
izTRAIL [ng/ml]
0 0.1 1 10 100 1000
si-Ctrl
si-CDK7
si-CDK9
si-FLT3
si-PHKG1
si-CDK14
si-HIPK1
si-HIPK2
si-PKA1
si-PKA2
si-CLK1
si-CLK2
si-CLK3
si-CLK4
si-JAk2
si-JAK3
si-PKCepsilon
si-CK2A2
si-LATS2
si-PKCetha
si-PKCtheta
si-Dyrk1a
si-Dyrk1b
si-MAP4K2
si-PKN1
si-ERK8
si-cMET
si-YSK4

B

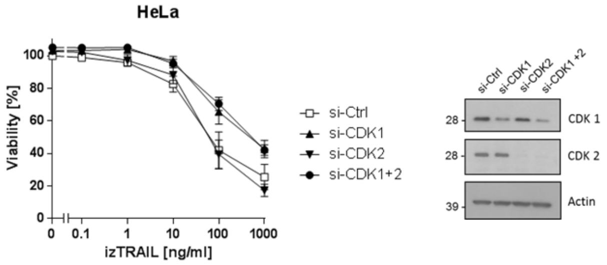

C

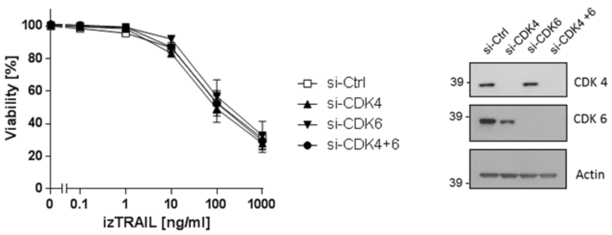

Figure S2 (a) HeLa cells were subjected to the indicated knockdowns for 48h and subsequently stimulated with the indicated concentrations of izTRAIL. Cell viability was quantified after 24h. (b) HeLa cells were transfected with siRNA targeting CDK1 and/or CDK2 for 48h and subsequently stimulated with the indicated concentrations of izTRAIL. Cell viability was quantified after 24h. (c) HeLa cells were transfected with siRNA targeting CDK4 and/or CDK6 for 48h and subsequently stimulated with the indicated concentrations of izTRAIL. Representative western blots are shown. Values represent means ± SEM of three independent experiments (b-c)

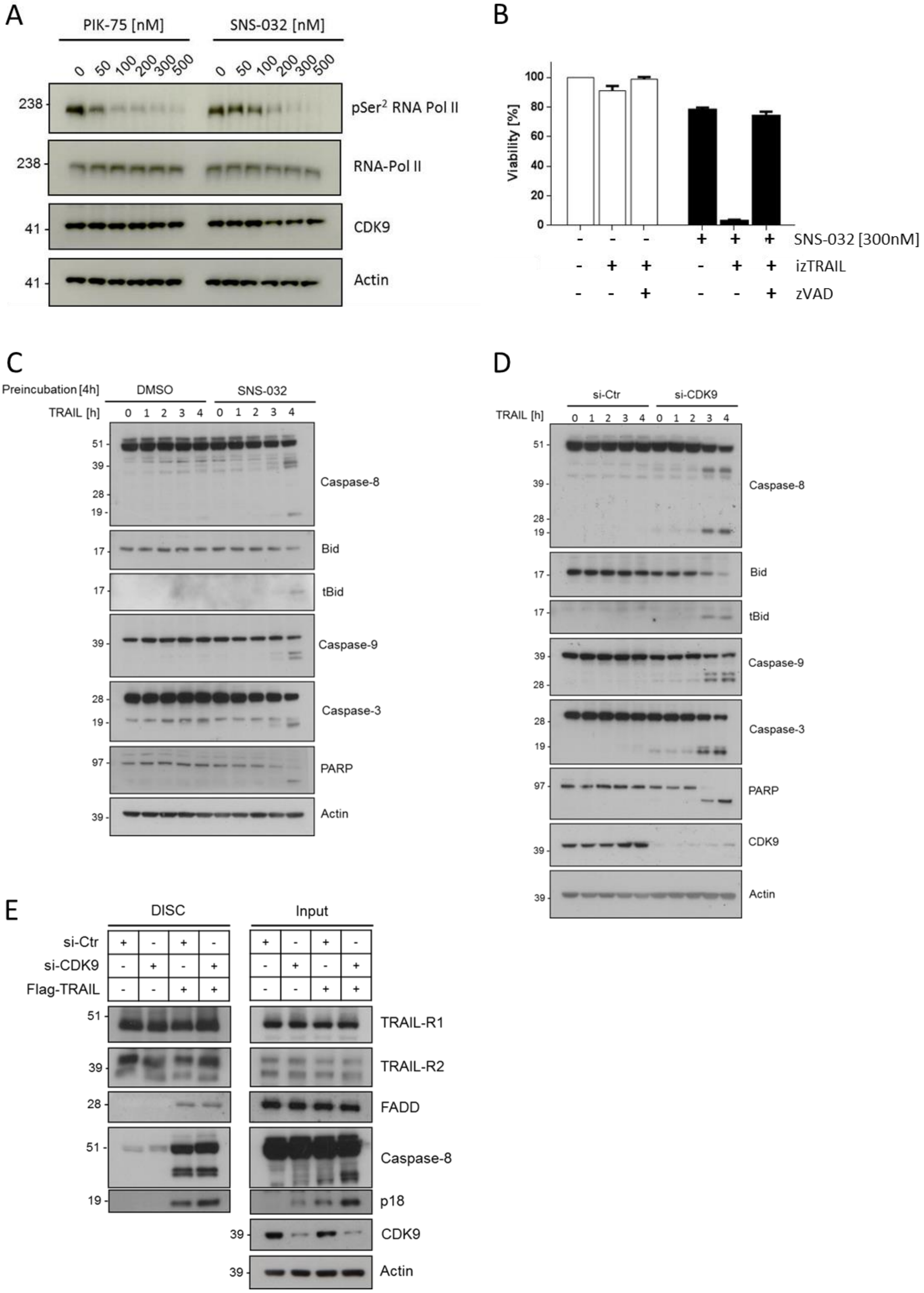
A
PIK-75 [nM]
SNS-032 [nM]
0 50 100 200 300 500
0 50 100 200 300 500
238
238
41
41
pSer2 RNA Pol II
RNA-Pol II
CDK9
Actin
B
Viability [%]
100
80
60
40
20
0
SNS-032 [300nM]
izTRAIL
zVAD
C
Preincubation [4h]
DMSO
SNS-032
TRAIL [h]
0 1 2 3 4 0 1 2 3 4
Caspase-8
Bid
tBid
Caspase-9
Caspase-3
PARP
Actin
D
si-Ctr
si-CDK9
TRAIL [h]
0 1 2 3 4 0 1 2 3 4
Caspase-8
Bid
tBid
Caspase-9
Caspase-3
PARP
CDK9
Actin
E
DISC
Input
si-Ctr
si-CDK9
Flag-TRAIL
TRAIL-R1
TRAIL-R2
FADD
Caspase-8
p18
CDK9
Actin

Figure S3 (a) HeLa cells were treated for 3h with PIK-75 or SNS-032 at the indicated concentrations. Cells were subsequently lysed and subjected to western blotting. One representative of two independent experiments is shown. (b) HeLa cells were preincubated with 300nM SNS-032 ± 20 μM zVAD for 1h and subsequently stimulated with 10 ng/ml izTRAIL. Cell viability was quantified after 24h. Values represent means ± SEM of three independent experiments (c) HeLa cells were preincubated with DMSO or SNS-032 [300 nM] for 4h and subsequently stimulated with izTRAIL [10 ng/ml] for the indicated times. Cells were lysed and subjected to western blotting. One representative of two independent experiments is shown. (d) HeLa cells were transfected with siRNA targeting CDK9 for 72h and subsequently stimulated with izTRAIL [10 ng/ml] for the indicated times. Cells were lysed and subjected to western blotting. One representative of two independent experiments is shown. (e) A549 cells were subjected to indicated knockdowns for 72h, stimulated with Flag-TRAIL (1µg/ml] for 1h and subsequently the TRAIL-DISC was analysed by western blotting. One representative of two independent experiments is shown.

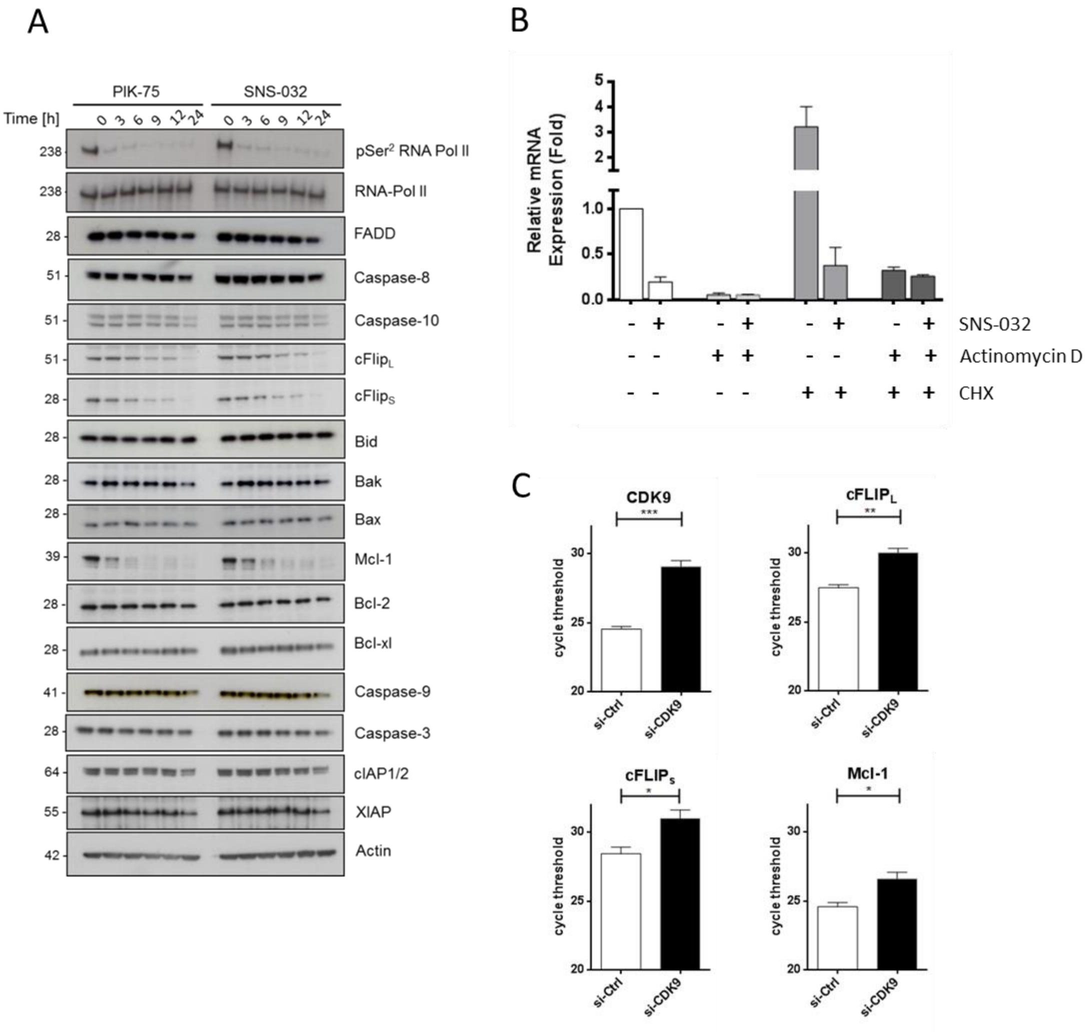

Figure S4 (a) HeLa cells were treated with PIK-75 (100 nM) or SNS-032 (300 nM) for the indicated times. Cells were lysed and subjected to western blotting. One representative of two independent experiments is shown. (b) A549 cells were preincubated with cycloheximide (CHX; 1 µg/ml) and/or actinomycin D (2 µg/ml) and/or SNS-032 (300 nM) as indicated. Mcl-1 mRNA expression was quantified by RT-PCR. Values represent means ± SEM of at least two independent experiments. (c) HeLa cells were transfected with siRNA targeting CDK9. After 72h mRNA of 500.000 cells per condition was isolated, cDNA was generated and expression of indicated target genes was quantified by RT-PCR. Cycle threshold for the indicated transcripts is shown. Values represent means ± SEM of three independent experiments. * $P < 0.05$; ** $P < 0.01$; *** $P < 0.001$ Student's t-test

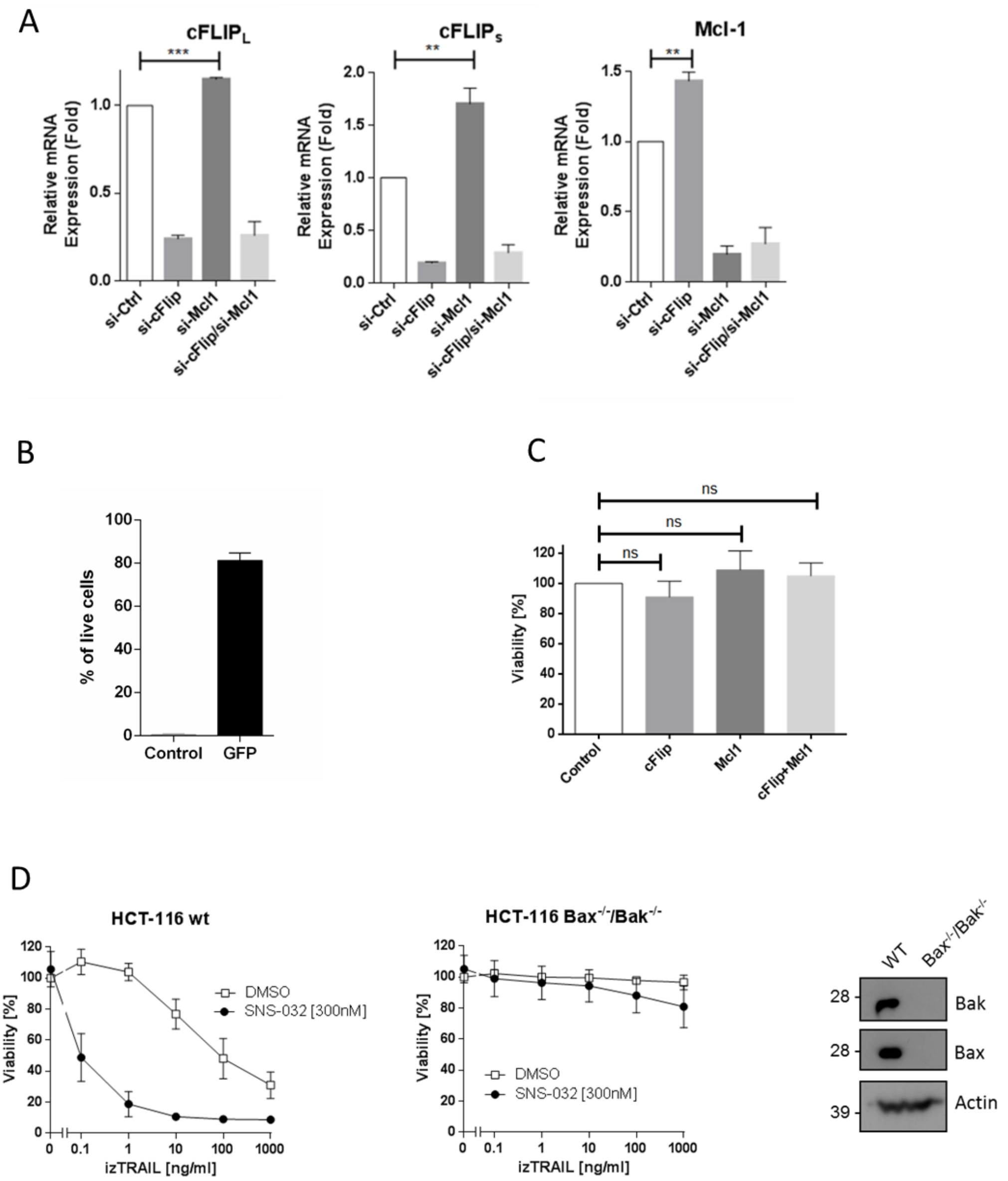

Figure S5 (a) HeLa cells were subjected to indicated knockdowns and mRNA expression of indicated target genes was quantified by RT-PCR relative to GAPDH. (b) HeLa cells were transfected with a GFP-containing plasmid and GFP positive cells were quantified by flow cytometry. (c) HeLa cells were transfected with expression plasmids for cFlip and/or Mcl-1 or empty vector control for and cell viability was quantified after48h. (d) $HCT116^{WT}$ or $HCT116^{Bax-/-/Bak-/-}$ cells were pre-incubated with SNS-032 [300 nM] and subsequently stimulated with izTRAIL at the indicated concentrations. Cell viability was quantified after 24h. Representative western blots to confirm Bax/Bak deficiency are shown. All values represent means ± SEM of three independent experiments. ns = not significant; ** $P < 0.01$; *** $P < 0.001$ Student's t-test

A

NSCLC cell line	KRAS	TP53
A549	■	
Calu-1	■	
H460	■	
H322		■
H522		■
H23	■	■
H441	■	■

B

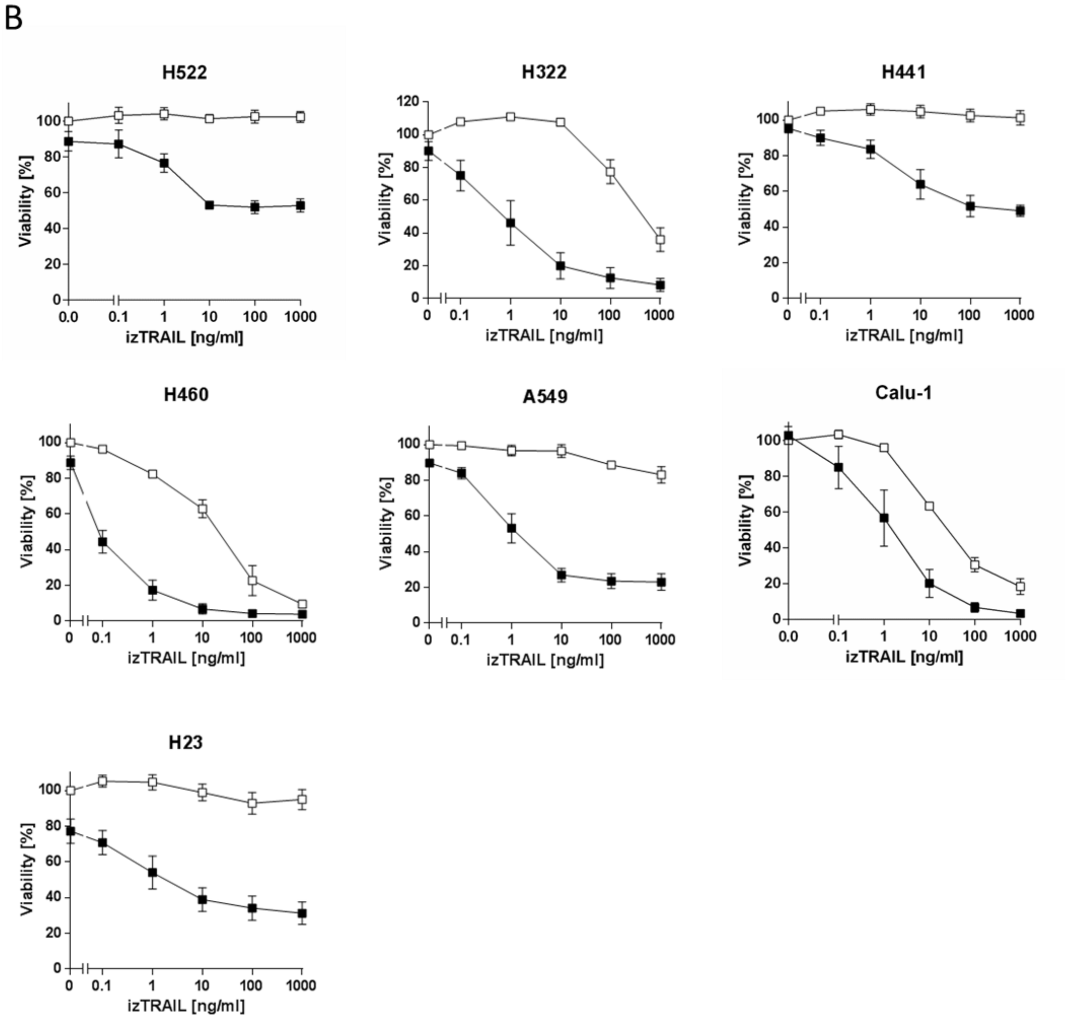

Figure S6 (a) Mutations in KRAS and/or p53 of cell lines included in the NSCLC panel (Figure 6a) are indicated by a black box. (b) Cell lines included in the NSCLC panel (Figure 6a) were preincubated with DMSO (open square) or SNS-032 [300 nM] (filled square) for 1h and subsequently stimulated with izTRAIL at the indicated concentrations. Cell viability was quantified after 24h. Values are means ± SEM of three independent experiments.

A

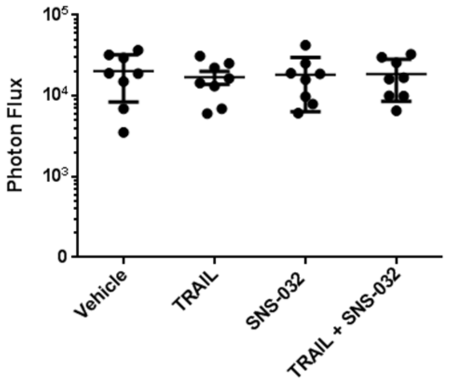

Figure S7 Tumor burden was quantified by bioluminescence imaging (Photon Flux) one week after injection of $2x10^6$ A549-Luc cells. Dots represent individual mice (n=8 per group) +/- SEM before treatment (after randomization).

Supplementary Literature

1. Knight ZA, Gonzalez B, Feldman ME, Zunder ER, Goldenberg DD, Williams O *et al.* A pharmacological map of the PI3-K family defines a role for p110alpha in insulin signaling. *Cell* 2006; 125(4): 733-47.

2. Jackson SP, Schoenwaelder SM, Goncalves I, Nesbitt WS, Yap CL, Wright CE *et al.* PI 3-kinase p110beta: a new target for antithrombotic therapy. *Nature medicine* 2005; 11(5): 507-14.

3. Pomel V, Klicic J, Covini D, Church DD, Shaw JP, Roulin K *et al.* Furan-2-ylmethylene thiazolidinediones as novel, potent, and selective inhibitors of phosphoinositide 3-kinase gamma. *Journal of medicinal chemistry* 2006; 49(13): 3857-71.

4. Folkes AJ, Ahmadi K, Alderton WK, Alix S, Baker SJ, Box G *et al.* The identification of 2-(1H-indazol-4-yl)-6-(4-methanesulfonyl-piperazin-1-ylmethyl)-4-morpholin-4-yl-t hieno[3,2-d]pyrimidine (GDC-0941) as a potent, selective, orally bioavailable inhibitor of class I PI3 kinase for the treatment of cancer. *Journal of medicinal chemistry* 2008; 51(18): 5522-32.

5. Jamieson S, Flanagan JU, Kolekar S, Buchanan C, Kendall JD, Lee WJ *et al.* A drug targeting only p110alpha can block phosphoinositide 3-kinase signalling and tumour growth in certain cell types. *The Biochemical journal* 2011; 438(1): 53-62.

6. Maira SM, Stauffer F, Brueggen J, Furet P, Schnell C, Fritsch C *et al.* Identification and characterization of NVP-BEZ235, a new orally available dual phosphatidylinositol 3-kinase/mammalian target of rapamycin inhibitor with potent in vivo antitumor activity. *Molecular cancer therapeutics* 2008; 7(7): 1851-63.
